# Comparing the quality of dying for patients with hematological malignancy and solid tumors: A bereavement study in Japan

**DOI:** 10.1002/cncr.70438

**Published:** 2026-05-01

**Authors:** Shohei Ikeda, Yusuke Hiratsuka, Yoko Nakazawa, Mitsunori Miyashita, Tatsuya Morita, Yasuyuki Okumura, Yoshiyuki Kizawa, Shohei Kawagoe, Hiroshi Yamamoto, Emi Takeuchi, Risa Yamazaki, Asao Ogawa

**Affiliations:** ^1^ Department of Palliative Medicine Takeda General Hospital Aizuwakamatsu Japan; ^2^ Department of Hematology Fukushima Medical University Aizu Medical Center Aizuwakamatsu Japan; ^3^ Department of Palliative Medicine Tohoku University Graduate School of Medicine Sendai Japan; ^4^ Division of Policy Evaluation Institute for Cancer Control National Cancer Center Chuo‐ku Japan; ^5^ Department of Palliative Nursing, Health Sciences Tohoku University Graduate School of Medicine Sendai Japan; ^6^ Department of Palliative and Supportive Care Seirei Mikatahara General Hospital Hamamatsu Japan; ^7^ Research Association for Community Health Hamamatsu Japan; ^8^ Initiative for Clinical Epidemiological Research Machida Japan; ^9^ Department of Palliative and Supportive Care Institute of Medicine Tsukuba Japan; ^10^ Aozora Clinic Matsudo Japan; ^11^ Department of Respiratory Medicine Tokyo Metropolitan Institute for Geriatrics and Gerontology Itabashi‐ku Japan; ^12^ Division of Quality Assurance Programs Institute for Cancer Control National Cancer Center Chuo‐ku Japan; ^13^ Department of Medical Psychology Graduate School of Medical Sciences Kitasato University Sagamihara Japan; ^14^ Division of Psycho‐Oncology Exploratory Oncology Research and Clinical Trial Center National Cancer Center Kashiwa Japan

**Keywords:** bereavement study, hematological malignancies, palliative care, quality of care, quality of dying

## Abstract

**Background:**

Although research on palliative care in hematological malignancies has increased, research examining quality of death (QOD) and quality of care (QOC) in this population remains limited. This study compared QOD and QOC between patients with hematological malignancies and those with solid tumors.

**Methods:**

The authors conducted a secondary analysis of a nationwide mortality follow‐up survey of bereaved family members in Japan (2017–2018). The study included 3575 decedents with hematological malignancies and 50,592 with solid tumors. Propensity score matching was performed to adjust for demographic and clinical characteristics. QOD and QOC were assessed using the Good Death Inventory (GDI) and the Care Evaluation Scale 2.0 (CES). Bivariate analyses compared the matched groups.

**Results:**

Overall, QOD and QOC were comparable between groups. However, among the GDI subdomains, patients with hematological malignancies had slightly lower scores for “good relationships with family” (mean difference, 0.2; 95% confidence interval [CI], 0.03–0.3) and “preparation for death” (mean difference, 0.2; 95% CI, 0.04–0.3). In addition, patients with hematological malignancies were less likely to die in palliative care units than those with solid tumors (mean difference, 3.9%; 95% CI, 0.4%–7.4%).

**Conclusions:**

Although overall quality measures were similar, specific QOD domains related to family relationships and preparation for death were slightly lower among patients with hematological malignancies. These findings may reflect limited opportunities for end‐of‐life discussions due to the unpredictable and rapidly progressive course of hematological malignancies. Enhancing communication about prognosis and goals of care and early integration of palliative care may improve end‐of‐life experiences.

## Graphical abstract

Quality of death (QOD) and quality of care (QOC) in hematological malignancies reflect disease‐specific contexts, emphasizing the need for tailored palliative care strategies.

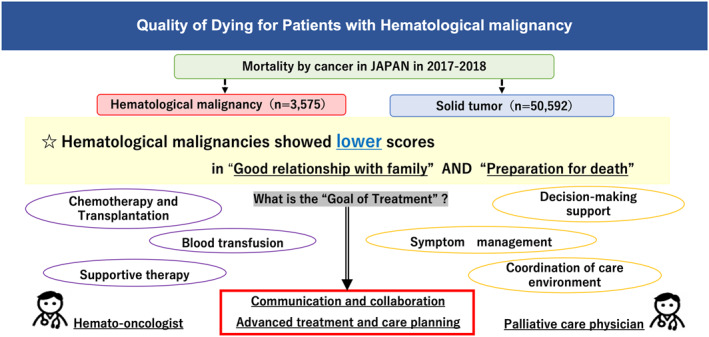

## INTRODUCTION

Palliative care is an essential component of comprehensive cancer treatment and aims to alleviate symptom burden and improve quality of life.^1^ Compared with patients with solid tumors, those with hematological malignancies often have a more unpredictable disease trajectory,^2^ require ongoing transfusion support,^3^, ^4^ experience complex infections,^5^ and face challenges in prognostication.^4^, ^6^, ^7^ They frequently continue intensive disease‐directed therapies until late in the course of illness.^8^ Given these complexities, patients with hematopoietic malignancies may develop an overly optimistic understanding of their prognosis despite substantial clinical uncertainty.^9^

Given these complex clinical features, patients with hematological malignancies often experience substantial symptom burdens. Nevertheless, timely integration of specialist palliative care remains limited. Palliative care services are underused in this population, and consultations frequently occur late in the disease trajectory. These findings underscore the need for improved integration of palliative care within hematology practice.^10^

These clinical challenges highlight the importance of maintaining high quality of care (QOC) and improving the quality of dying (QOD) for patients with hematological malignancies and their families. Although patient self‐report is considered the gold standard for evaluating care, its feasibility is often limited in end‐of‐life research due to clinical deterioration and cognitive impairment. Therefore, bereaved family members’ evaluations have become an established approach for assessing the quality of hospice and palliative care.^11^, ^12^ Most previous studies on QOD and QOC have focused on patients with solid tumors,^13^, ^14^ leaving limited evidence in hematological malignancies.

We hypothesized that understanding these differences could lead to improvements in the quality of end‐of‐life care. Therefore, this study aimed to compare the QOD and QOC of patients with hematological malignancies and those with solid tumors.

## MATERIALS AND METHODS

### Participants

This study was a secondary analysis of a cross‐sectional, nationwide mortality follow‐up survey. The original study aimed to examine family‐reported outcomes of end‐of‐life care by location and cause of death among patients with five common causes of death (cancer, heart disease, stroke syndrome, pneumonia, and renal failure) in Japan.^15^

The inclusion criteria for decedents were as follows: 1) older than 20 years of age at death, 2) death in either a hospital, long‐term care facility, or at home, and 3) patients were diagnosed with locally extensive cancer or cancer with metastases. Therefore, only data from cancer patients and their bereaved family members were extracted for this study.

### Data collection

We used the most recent available mortality data from 2017 and 2018. The 2017 target population included patients who died from any of the five leading causes of death in Japan (cancer, heart disease, stroke syndrome, pneumonia, and renal failure), excluding natural and accidental deaths. In February 2019 and February 2020, two questionnaires were distributed by mail to patients’ addresses. Between 13 and 25 months after the patient’s death, we asked bereaved family members about the decedent’s end‐of‐life experiences.

We collected information about QOC and QOD from respondents’ data. Data on the following decedent characteristics were obtained: primary cancer site, age, sex, living with family (yes or no), time from diagnosis to death (≤24 hours, ≤1 week, ≤1 month, ≤3 months, ≤1 year, ≤5 years, ≤10 years, or >10 years), difficulty with activities of daily living during the last month (independent, partly independent, or almost completely independent), difficulty with communication during the last month (no difficulty, some difficulty, mostly unable to communicate, or unable to communicate at all), coexistence of dementia (present or absent), specialized palliative care intervention (received or not received) and home medical care during the last 6 months (received or not received). We defined a specialized palliative care intervention as one provided by a palliative care team, a palliative care unit, a palliative care outpatient clinic, or a home hospice care. Data about primary cancer site, age, and sex were calculated using death certificate data for vital statistics.

We collected the following data about respondents: age, sex, and relationship to decedent (spouse, child, child‐in‐law, parent, relative, or others).

### Outcomes

The Japanese version of the Good Death Inventory (GDI) was used to evaluate QOD^16^ in the last month of life. The GDI core items comprise 18 domains representing concepts that are considered important for a good death, including “Physical and psychological comfort” and “Being respected as an individual.” The GDI has adequate reliability and validity and has been used in large‐scale quantitative studies.^17^, ^18^, ^19^, ^20^, ^21^ Family members were asked to rate the decedents’ experiences in the last month of life on a 7‐point Likert scale (ranging from 1: strongly disagree to 7: strongly agree). The total score was obtained by summing the 18 domain scores and converting to a maximum of 126. Higher scores indicated better QOD.

The Japanese version of the Care Evaluation Scale 2.0 (CES) was used to evaluate QOC^22^ at the decedent’s place of death. The short version of the CES consists of 10 domains representing concepts that are important to end‐of‐life care, including “Physical care by nurse” and “Psycho‐existential care.” The CES has shown adequate reliability and validity, and has been used in large‐scale quantitative studies.^17^, ^18^, ^19^, ^20^, ^21^ Family members were asked to rate QOC at the decedent’s last place of care using a 6‐point Likert scale (ranging from 1: strongly disagree to 6: strongly agree). The total score was obtained by summing the 10 domain scores and converting to a 100‐point scale. Higher scores indicated better QOC. In addition, we asked the bereaved family members to rate their overall satisfaction with the last place of care using a 6‐point Likert scale (1: strongly disagree to 6: strongly agree).

Drawing on a review of the literature, we obtained the following data to examine family‐reported outcomes of end‐of‐life care^17^, ^18^, ^23^, ^24^: occurrence of death in a palliative care unit, ambulance use during the last month, performance of cardiopulmonary resuscitation, discussion about performing cardiopulmonary resuscitation with the attending physician, end‐of‐life discussion between patient and family during the last month, and end‐of‐life discussion between family and attending physician during the last month.

### Statistical analysis

First, decedents were categorized into “hematological malignancy” and “solid tumor” groups. We defined the hematological malignancy group as decedents with a diagnosis of leukemia, malignant lymphoma, or other hematological malignancies.

Second, the baseline and clinical characteristics of the decedents and respondents were summarized using descriptive analysis.

Third, we used propensity scores to compare the hematological malignancy and solid tumor groups. The predicted probability of hematological malignancies was calculated by fitting a logistic regression model, using the following 12 covariates that may affect QOD or QOC according to our review of the literature^17^, ^18^, ^23^, ^24^: age of decedents and respondents, sex of decedents and respondents, family living with decedents, time from diagnosis to death of decedents, difficulty with activities of daily living during decedents’ last month, difficulty with communication during decedents’ last month, coexistence of dementia in decedents, specialized palliative care intervention provided to decedents by palliative care physicians (palliative care team, palliative care unit, palliative care outpatient clinic, or home hospice care), home medical care during decedents’ last 6 months, and respondents’ relationship to the decedents. We rigorously adjusted the eight covariates with propensity score matching using the following algorithm: 1:1 optimal matching with a caliper width of 0.2 times the standard deviation of the logit of the propensity score and no replacement. We considered a standardized mean difference <10% as indicating good covariate balance.^25^, ^26^

Fourth, we performed bivariate analysis to compare differences in QOD, QOC, respondent outcome (overall care satisfaction), and family‐reported outcomes of end‐of‐life care between the hematological malignancy group and the solid tumor group. We performed a complete case analysis. We calculated the differences in means or percentages between the two groups with a 95% confidence interval.

All analyses were performed using JMP version 17.0 for Windows (SAS, Cary, North Carolina, USA). Statistical significance was set at *p* < .05.

### Ethics

All study procedures were approved by the National Cancer Center Japan institutional review board (reference number 2017‐346; date of approval June 5, 2018). The study was conducted in accordance with the ethical standards of the Declaration of Helsinki (as revised in 2013). This study followed the ethical guidelines for medical and health research involving human subjects presented by the Japanese Ministry of Health, Labour and Welfare. For bereavement studies of this type, Japanese law does not require individual informed consent from participants. Consent to participate in the study was given by respondents who completed and returned the questionnaires.

## RESULTS

### Participant characteristics

**FIGURE 1:**
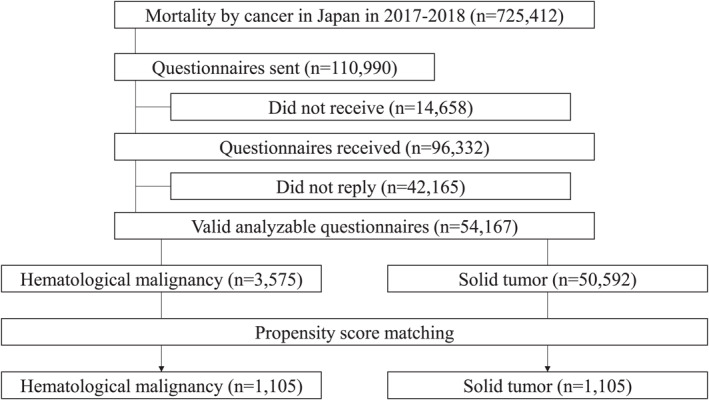
Participants selection flow.

We mailed questionnaires to 110,990 family members. Of these, 96,332 family members received the questionnaires, of which 54,167 provided valid responses (response rate: 56.2%). Thus, 54,167 responses were ultimately analyzed (Figure 1). Decedents’ and respondents’ characteristics are summarized in Table 1. The study decedents included 3575 decedents (6.6%) with hematological malignancy and 50,592 decedents (93.4%) with solid tumors. The types of hematological malignancies were as follows: leukemia in 1195 decedents (33.4%), malignant lymphoma in 1743 decedents (48.8%), and other hematological malignancies in 637 decedents (17.8%). There were 32,098 male decedents (59.3%) and 22,069 female decedents (40.7%). There were 18,525 male respondents (34.6%) and 35,032 female respondents (65.4%). The mean age of the decedents was 78.0 years (SD, 11.4 years) and that of the respondents was 65.0 years (SD, 11.9 years).

**TABLE 1:** Characteristics of participants.

Characteristics	All (*n* = 54,167)	Hematological malignancy group (*n* = 3575)	Solid tumor group (*n* = 50,592)	Missing value
Decedents				
Age (years)^a^	78.0 ± 11.4	78.1 ± 11.7	78.0 ± 11.4	0
Sex^a^				0
Male	32,098 (59.3)	2023 (56.6)	30,075 (59.5)	
Female	22,069 (40.7)	1552 (43.4)	20,517 (40.6)	
Living with family				811
Yes	47,016 (88.1)	3119 (88.5)	43,897 (88.1)	
No	6340 (11.9)	405 (11.5)	5935 (11.9)	
Time from diagnosis to death				1255
≤24 hours	303 (0.6)	42 (1.2)	261 (0.5)	
≤1 week	855 (1.6)	117 (3.3)	738 (1.5)	
≤1 month	3695 (7.0)	338 (9.6)	3357 (6.8)	
≤3 months	7737 (14.6)	499 (14.2)	7238 (14.7)	
≤1 year	15,926 (30.1)	987 (28.1)	14,939 (30.2)	
≤5 years	18,314 (34.6)	1055 (30.1)	17,259 (34.9)	
≤10 years	3895 (7.4)	331 (9.4)	3564 (7.2)	
>10 years	2187 (4.1)	139 (4.0)	2048 (4.2)	
Difficulty with activities of daily living during the last month				594
Independent	11,095 (20.7)	881 (25.0)	10,214 (20.4)	
Partially	18,542 (34.6)	1132 (32.1)	17,410 (34.8)	
Almost all	23,936 (44.7)	1513 (42.9)	22,423 (44.8)	
Difficulty with communication during the last month				560
No difficulty	30,513 (56.9)	2082 (58.8)	28,431 (56.8)	
Some difficulty	17,129 (32.0)	1024 (28.9)	16,105 (32.2)	
Mostly unable to communicate	4001 (7.5)	282 (8.0)	3719 (7.4)	
Unable to communicate at all	1964 (3.7)	155 (4.4)	1809 (3.6)	
Coexistence of dementia				1620
Present	7218 (13.7)	473 (13.6)	6745 (13.7)	
Absent	45,329 (86.3)	2996 (86.4)	42,333 (86.3)	
Specialized palliative care intervention				6974
Received	23,313 (49.4)	1121 (37.0)	22,192 (50.3)	
Not received	23,880 (50.6)	1912 (63.0)	21,968 (49.8)	
Home medical care during the last 6 months				6382
Received	20,213 (42.3)	973 (31.7)	19,240 (43.0)	
Not received	27,572 (57.7)	2096 (68.3)	25,476 (57.0)	
Respondents				
Age (years)	65.0 ± 11.9	64.74 ± 12.17	64.99 ± 11.84	689
Sex				610
Male	18,525 (34.6)	1228 (34.8)	17,297 (34.6)	
Female	35,032 (65.4)	2304 (65.2)	32,728 (65.4)	
Relationship to the decedent				670
Spouse	23,882 (44.6)	1509 (42.8)	22,373 (44.8)	
Child	21,519 (40.2)	1474 (41.8)	20,045 (40.1)	
Child‐in‐law	4393 (8.2)	298 (8.5)	4095 (8.2)	
Parent	1172 (2.2)	86 (2.4)	1086 (2.2)	
Relative	2216 (4.1)	139 (4.0)	2077 (4.2)	
Other	315 (0.6)	17 (0.5)	298 (0.6)	

*Note*: Data are presented as mean (standard deviation), No. (%), or median (range).

^a^ Data were calculated using death certificate data of vital statistics.

### Propensity score matching balance

Table 2 shows the balance of covariates. After matching, the covariates were well balanced between the hematological malignancy and solid tumor groups, within 10% of the standardized mean difference.

**TABLE 2:** Covariate balance before and after propensity score matching.

Covariates	Unmatched			Matched		
	Hematological malignancy group (*n* = 3575)	Solid tumor group (*n* = 50,592)	SMD	Hematological malignancy group (*n* = 1105)	Solid tumor group (*n* = 1105)	SMD
Decedents						
Age, years, mean (SD)	78.1 (11.7)	78.0 (11.4)	0.01	78.2 (11.7)	78.2 (11.0)	<0.01
Sex (male) (%)	56.6	59.5	0.06	55.9	53.6	0.05
Living with family (%)	88.5	88.1	0.01	89.5	80.1	0.02
Time from diagnosis to death (%)						
≤24 hours	1.2	0.5	0.08	0.9	1.4	0.04
≤1 week	3.3	1.5	0.12	1.9	1.9	0
≤1 month	9.6	6.8	0.10	8.0	8.2	<0.01
≤3 months	14.2	14.7	0.01	14.2	14.0	0.01
≤1 year	28.1	30.2	0.05	29.1	28.8	<0.01
≤5 years	30.1	34.9	0.10	31.7	30.0	0.04
≤10 years	9.4	7.2	0.08	9.8	10.8	0.03
>10 years	4.0	4.2	0.01	4.4	5.0	0.03
Difficulty with activities of daily living during the last month (%)						
Independent	25.0	20.4	0.11	25.0	24.2	0.02
Partially	32.1	34.8	0.06	34.7	33.5	0.03
Almost all	42.9	44.8	0.04	40.4	42.4	0.04
Difficulty with communication during the last month (%)						
No difficulty	58.8	56.8	0.04	62.3	62.9	0.01
Some difficulty	28.9	32.2	0.07	30.5	29.6	0.02
Mostly unable to communicate	8.0	7.4	0.02	5.7	5.8	<0.01
Unable to communicate at all	4.4	3.6	0.04	1.5	1.7	0.02
Coexistence of dementia (%)	13.6	13.7	<0.01	11.1	12.0	0.03
Specialized palliative care intervention, No. (%)	37.0	50.3	0.27	38.3	39.6	0.03
Home medical care during the last 6 months (%)	31.7	43.0	0.24	31.7	32.9	0.03
Respondents						
Age, years, mean (SD)	64.7 (12.2)	65.0 (11.8)	0.02	63.5 (11.5)	63.3 (11.2)	<0.02
Sex (male)	34.8	34.6	<0.01	35.7	40.1	0.09
Relationship to the decedent						
Spouse	42.8	44.8	0.04	40.6	38.4	0.05
Child	41.8	40.1	0.03	45.2	45.9	0.01
Child‐in‐law	8.5	8.2	0.01	8.4	9.6	0.04
Parent	2.4	2.2	0.01	2.7	3.0	0.02
Relative	4.0	4.2	0.01	2.8	2.7	<0.01
Other	0.5	0.6	0.01	0.3	0.5	0.03

Abbreviations: SD, standard deviation; SMD, standardized mean difference.

Differences in QOC, QOD, respondents’ outcomes, and family‐reported outcomes of end‐of‐life care between the hematological malignancy and solid tumor groups

Table 3 shows the differences in QOD, QOC, respondents’ outcomes, and family‐reported outcomes of end‐of‐life care between the hematological malignancy group and the solid tumor group. For the QOD domains, “Good relationship with family” and “Preparation for death” showed slightly higher scores in the solid tumor group compared with the hematological malignancy group. The mean score for “Good relationships with family” was 5.1 (SD, 1.5) in the solid tumor group and 4.9 (SD, 1.6) in the hematological malignancy group (mean difference, 0.2; 95% confidence interval [CI], 0.03–0.3). Similarly, the mean score for “Preparation for death” was 4.5 (SD, 1.6) in the solid tumor group and 4.4 (SD, 1.7) in the hematological malignancy group (mean difference, 0.2; 95% CI, 0.04–0.3). For the QOC domains, “Physical care by nurse,” “Physician’s explanation to the patient,” and “Physician’s explanation to the family” showed slightly higher scores in the hematological malignancy group. The mean score for “Physical care by nurse” was 4.9 (SD, 0.9) in the solid tumor group and 4.9 (SD, 0.9) in the hematological malignancy group (mean difference, −0.09; 95% CI, −0.20 to −0.01). The mean score for “Physician’s explanation to the patient” was 4.7 (SD, 1.2) in the solid tumor group and 4.8 (SD, 1.1) in the hematological malignancy group (mean difference, −0.1; 95% CI, −0.2 to −0.02). Similarly, the mean score for “Physician’s explanation to the family” was 4.8 (SD, 1.1) and 4.9 (SD, 1.1), respectively (mean difference, −0.1; 95% CI, −0.2 to −0.02). Regarding family‐reported outcomes of end‐of‐life care, “Death in palliative care unit” was more frequent in the solid tumor group. The percentage of “Death in palliative care unit” was 21.3% in the solid tumor group and 17.4% in the hematological malignancy group (mean difference, 3.9%; 95% CI, 0.4%–7.4%).

**TABLE 3:** Differences between the hematological malignancy group and the solid tumor group after propensity score matching.

Scales	Hematological malignancy group (*n* = 1105)	Solid tumor group (*n* = 1105)	Mean difference (95% CI)^a^	Missing value
GDI (mean ± SD)				
Physical and psychological comfort	4.2 ± 1.6	4.1 ± 1.7	–0.1 (–0.2 to 0.03)	0
Dying in a favorite place	4.7 ± 1.9	4.9 ± 1.9	0.2 (–0.003 to 0.3)	0
Maintaining hope and pleasure	4.0 ± 1.8	4.0 ± 1.8	0.01 (–0.1 to 0.2)	0
Good relationship with medical staff	5.3 ± 1.4	5.3 ± 1.4	–0.03 (–0.1 to 0.09)	0
Not being a burden to others	3.1 ± 1.5	3.1 ± 1.5	0.01 (–0.1 to 0.1)	0
Good relationship with family	4.9 ± 1.6	5.1 ± 1.5	0.2 (0.03–0.3)	0
Independence	3.4 ± 2.0	3.3 ± 1.9	–0.1 (–0.3 to 0.05)	0
Environmental comfort	5.0 ± 1.4	5.1 ± 1.5	0.09 (–0.03 to 0.2)	0
Being respected as an individual	5.7 ± 1.2	5.7 ± 1.2	0.05 (–0.05 to 0.1)	0
Life completion	4.6 ± 1.8	4.7 ± 1.8	0.08 (–0.07 to 0.2)	0
Receiving enough treatment	4.7 ± 1.6	4.6 ± 1.6	–0.12 (–0.3 to 0.01)	0
Natural death	4.7 ± 1.7	4.9 ± 1.6	0.1 (–0.02 to 0.3)	0
Preparation for death	4.4 ± 1.7	4.5 ± 1.6	0.2 (0.04–0.3)	0
Control over the future	4.1 ± 1.7	4.2 ± 1.7	0.07 (–0.08 to 0.2)	0
Unawareness of death	3.6 ± 1.6	3.5 ± 1.6	–0.04 (–0.2 to 0.1)	0
Pride and beauty	2.8 ± 1.4	2.8 ± 1.5	0.04 (–0.08 to 0.2)	0
Feeling that one’s life is worth living	4.6 ± 1.4	4.5 ± 1.4	–0.05 (–0.2 to 0.07)	0
Religious and spiritual comfort	2.7 ± 1.8	2.7 ± 1.8	0.005 (–0.1 to 0.2)	0
Total score	76.4 ± 16.4	76.9 ± 16.5	0.5 (–0.9 to 1.9)	0
CES (mean ± SD)				
Physical care by physician	4.9 ± 1.0	4.8 ± 1.0	–0.04 (–0.1 to 0.04)	32
Physical care by nurse	4.9 ± 0.9	4.9 ± 0.9	–0.09 (–0.2 to –0.01)	40
Psycho‐existential care	4.8 ± 1.0	4.8 ± 1.0	–0.04 (–0.1 to 0.04)	37
Physician’s explanation to the patient	4.8 ± 1.1	4.7 ± 1.2	–0.1 (–0.2 to –0.02)	36
Physician’s explanation to the family	4.9 ± 1.1	4.8 ± 1.1	–0.1 (–0.2 to –0.02)	18
Environment	4.7 ± 1.0	4.7 ± 1.0	0.02 (–0.07 to 0.1)	29
Cost	4.7 ± 0.9	4.7 ± 0.9	–0.05 (–0.1 to 0.03)	137
Consideration of family health	4.5 ± 1.1	4.5 ± 1.1	–0.06 (–0.2 to 0.03)	106
Availability	4.9 ± 1.1	4.9 ± 1.0	–0.005 (–0.09 to 0.08)	105
Coordination and consistency	4.8 ± 1.0	4.8 ± 1.0	–0.05 (–0.1 to 0.03)	59
Total score	48.2 ± 7.7	47.6 ± 7.5	–0.5 (–1.2 to 0.2)	393
Overall care satisfaction (mean ± SD)	4.4 ± 1.3	4.4 ± 1.3	–0.05 (–0.2 to 0.06)	81
Death in palliative care unit	168 (17.4)	211 (21.3)	3.9 (0.4–7.4)	251
Ambulance use during the last month	168 (15.4)	175 (16.0)	–0.6 (–3.7 to 2.4)	23
Discussing where to care with attending physician	489 (50.8)	530 (55.3)	4.4 (–0.03 to 8.9)	289
Performing CPR	131 (12.7)	108 (10.5)	2.2 (–0.5 to 5.0)	149
Discussing about performing CPR with attending physician	493 (67.0)	451 (63.9)	–3.1 (–8.0 to 1.8)	768
End‐of‐life discussion between patient and family during the last month	550 (55.1)	554 (55.6)	0.5 (–3.8 to 4.9)	216
End‐of‐life discussion between family and attending physician during the last month	706 (73.4)	688 (72.9)	–0.6 (–4.5 to 3.5)	304

Abbreviations: CES, Care Evaluation Scale; CI, confidence interval; CPR, cardiopulmonary resuscitation; GDI, Good Death Inventory; SD, standard deviation.

^a^ Differences are indicated as or percentage differences or differences in means.

## DISCUSSION

In this study, we sought to elucidate the differences in QOD and QOC between patients with hematological malignancies and those with solid tumors. Our findings indicate that QOD and QOC were comparable between patients with solid and those with hematological malignancies. However, there were small differences in individual items from overall scales.

Patients with hematological malignancies showed lower GDI scores than those with solid tumors for “Good relationship with family” and “Preparation for death.” In contrast, although the differences were not statistically significant, scores for “Independence” and “Receiving enough treatment” tended to be higher. It is likely that the difference in illness trajectories between patients with hematological malignancies and those with solid tumors may explain these results. A previous study identified three distinct patterns of illness trajectories, and it has been noted that diseases with aggressive malignancy (e.g., leukemia and highly aggressive lymphoma) tend to progress rapidly over a short period.^2^ This rapid progression may have led to inadequate preparation for death and a poorer relationship with the family. Furthermore, in contrast to solid tumor treatment, reduced‐dose palliative chemotherapy effectively alleviates both physical and mental symptoms in hematological tumors. Several studies have identified hematological malignancy as a key factor in the administration of chemotherapy immediately before death.^27^, ^28^ Nevertheless, even for patients with hematological malignancies, continuing chemotherapy indefinitely until the end of life is not ideal. End‐stage chemotherapy may cultivate an overly optimistic perception for prognosis, potentially leading to the pursuit of more invasive treatment options, hindering appropriate preparation for death, and compromising fair decision‐making.^9^ It is essential for health care professionals to establish appropriate treatment goals through clear and compassionate communication.^29^ The implementation of end‐of‐life care consistent with patient wishes is associated with family‐reported good deaths.^30^ Repeated and ongoing conversations about both best‐ and worst‐case scenarios during daily clinical care help patients and families prepare for what lies ahead. As treatments become more advanced and specialized, it is increasingly important to clarify treatment goals for each condition. Further research into the role of palliative chemotherapy is also needed.^31^, ^32^

Regarding QOC, no significant differences in overall satisfaction were observed. The hematological malignancy group showed slightly higher ratings than the solid tumor group on the following areas: “Physical care by nurses” and “Physician’s explanation to the patient and family.” Hematological malignancy is frequently managed at the same medical facility from initial diagnosis to treatment completion and ultimately to the terminal stage, because of the use of high‐risk, high‐reward therapies, frequent transfusions to manage cytopenia,^3^, ^4^ and the complexity of managing infections.^5^ Furthermore, a large proportion of hematologic malignancy patients need frequent outpatient follow‐up visits because of rapid disease progression.^2^ This frequent contact with health care providers may offer opportunities for detailed discussions about the patient’s condition.

We found that the mortality rate in palliative care units was lower in patients with hematological malignancy than in those with solid tumors. Patients with hematological malignancy frequently manifest symptoms that are as severe as those experienced by patients with solid tumors. This highlights the urgent need for palliative care.^10^, ^33^, ^34^ Although hematologists recognize the importance of palliative care,^35^, ^36^ substantial barriers remain to its implementation, including a shortage of dedicated teams, limited understanding, and communication challenges.^37^, ^38^ Thus, patients with hematological malignancy receive palliative care later and for a shorter duration than those with solid tumors, and have limited access to hospice care.^39^, ^40^ Early integration of palliative care alongside active treatment for patients with advanced hematological malignancy is needed to optimize the utilization of palliative care.^41^, ^42^

The strengths of this study include its large‐scale, nationwide design. To the best of our knowledge, this is the first large‐scale study to directly investigate QOD and QOC among patients with hematological malignancies and compare these outcomes with those of patients with solid tumors. However, there are also some limitations. First, the findings may not be generalizable to decedents outside Japan because of Japan’s unique medical service system and cultural background. Second, the survey response rate was 56.2%, and it is possible that nonresponse bias affected the results. Recall bias may also have been present because the responses from bereaved family members were collected 13–25 months after the patients’ deaths. Thus, the presence of queries or uncertainties related to symptom assessment domains may have influenced reported symptom burden. Third, the differences in QOD, QOC of decedents, and bereaved family members' outcomes between the two groups were minimal, although statistically significant differences were identified. Consequently, the clinical significance of these variations remains uncertain. Fourth, another potential limitation is the timing of data collection. This study was conducted using data from 2017 and 2018, which coincided with the period when immunotherapy and molecular targeted therapies were becoming increasingly integrated into routine cancer care. The rapid expansion of immune checkpoint inhibitors and other novel systemic therapies has altered survival trajectories and patterns of care for many cancer types, including both solid tumors and hematological malignancies.^43^ Evolving treatment paradigms may influence patients’ quality of dying, and QOD and QOC experiences may differ in more recent cohorts. Further research using data from the era of widespread immunotherapy and targeted therapy use would be valuable to assess whether the observed associations persist in the current treatment landscape. Finally, hematological malignancies are a diverse group of disease with many different subtypes.^44^, ^45^, ^46^ In this study, we evaluated these subtypes collectively and did not perform analyses according to individual disease categories. A more comprehensive analysis would be advantageous because of the variability in treatment approaches and objectives according to disease type, patient age, and place of care setting.

In conclusion, overall quality of care and quality of dying were similar between the two groups. QOD domains related to family relationships and preparation for death were slightly lower among patients with hematological malignancies. Differences in individual evaluation items highlight the need for a specialized approach to patients with hematological malignancies. These findings may be attributable to limited opportunities for end‐of‐life discussions in the context of the often unpredictable and rapidly progressive clinical trajectory of hematological malignancies. Enhancing communication regarding prognosis and treatment goals, together with early integration of palliative care, may further improve the quality of end‐of‐life experiences in this population.

## AUTHOR CONTRIBUTIONS

**Shohei Ikeda**: Conceptualization; data curation; writing–original draft; writing–review and editing. **Yusuke Hiratsuka**: Conceptualization; data curation; formal analysis; investigation; methodology; writing–original draft; writing–review and editing. **Yoko Nakazawa**: Investigation writing–review and editing. **Mitsunori Miyashita**: Investigation; writing–review and editing. **Tatsuya Morita**: Investigation; writing–review and editing. **Yasuyuki Okumura**: Data curation; formal analysis; investigation; writing–review and editing. **Yoshiyuki Kizawa**: Investigation; writing–review and editing. **Shohei Kawagoe**: Investigation; writing–review and editing. **Hiroshi Yamamoto**: Investigation; writing–review and editing. **Emi Takeuchi**: Investigation; writing–review and editing. **Risa Yamazaki**: Investigation; writing–review and editing. **Asao Ogawa**: Investigation; methodology; writing–review and editing; project administration.

## CONFLICT OF INTEREST STATEMENT

Shohei Kawagoe reports participation as a fiduciary officer for Aozora Clinic. Yoshiyuki Kizawa reports grant and/or contract funding from Chugai Pharmaceutical Co, Ltd and Shionogi, Inc. Yasuyuki Okumura reports fees for professional activities from Keio University and the National Cancer Center. Hiroshi Yamamoto reports fees for lectures from AstraZeneca, Bristol‐Myers Squibb, Boehringer Ingelheim Japan, Kyowa Kirin Co, Ltd, Daiichi Sankyo Co, Ltd, Sanofi, Life Technologies Japan Ltd, GlaxoSmithKline, Chugai Pharmaceutical Co, Ltd, Taiho Pharmaceutical Co, Ltd, Nippon Kayaku Co, Ltd, MSD, Teijin Healthcare Co, Ltd, Insmed, and Kyorin Pharmaceutical Co, Ltd. The other authors declare no conflicts of interest.

## Data Availability

The data that support the findings of this study are available on request from the corresponding author. The data are not publicly available due to privacy or ethical restrictions.
